# Mucormycosis: A Case Series

**DOI:** 10.7759/cureus.41375

**Published:** 2023-07-04

**Authors:** Jo Yen Yong, Kai Li Chong, Wee Fu Gan, Nor Zaila Zaidan

**Affiliations:** 1 Infectious Diseases, Hospital Melaka, Melaka, MYS; 2 Medicine, Hospital Melaka, Melaka, MYS

**Keywords:** immunocompromised, antifungal, case report, mucorales, mucormycosis

## Abstract

Mucormycosis is a potentially life-threatening invasive fungal infection caused by diverse fungal organisms in the order Mucorales. Traditional risk factors of mucormycosis include poorly controlled diabetes, hematological malignancies such as leukemia and lymphoma, and post-transplant patients, with rhino-orbito-cerebral and pulmonary mucormycosis as common manifestations. We report four cases of mucormycosis precipitated by classical as well as atypical risk factors, with common sites of infection such as pulmonary and rhino-orbital to rare manifestations such as peritoneal mucormycosis. Diagnoses were confirmed by either a histopathological sample or a positive culture. Only one patient had concomitant positive culture and histopathology results. Low culture positivity rate has delayed the diagnosis of two cases. First-line antifungal therapy was limited to amphotericin B deoxycholate in three cases due to financial cost, but all patients responded to the treatment. There were two mortalities, but both were unrelated to disease progression. All cases had source control done, except for the patient with pulmonary mucormycosis, due to poor lung reserve which refrained him from surgery. With emerging evidence of local therapies for endobronchial lesions, they potentially serve as an alternative for patients who are not suitable for operation. This case series also aims to contribute to the local epidemiology of mucormycosis, highlights the importance of early diagnosis, and draws attention from stakeholders to the challenges faced in managing this life-threatening infection.

## Introduction

Mucormycosis is a severe infection and is frequently fatal unless diagnosed early and treated aggressively. Diabetes mellitus is the most common risk factor in the Asian continent, whereas haematological malignancies and transplantation are the major risk factors in developed countries such as Europe and the United States [1,2]. The rise in mucormycosis incidence has been observed globally [3], and our centre also experienced a surge in cases in the past two years. However, the epidemiology of mucormycosis in Malaysia is not well described with only isolated case reports available [4,5]. We present a total of four mucormycosis cases, ranging from common to rare presentations. The first case was a pulmonary mucormycosis in the acquired immunodeficiency syndrome (AIDS) population, which was extremely rare as opposed to traditional risk factors, and the risk of infection was augmented by repetitive corticosteroid exposure due to chronic obstructive pulmonary disease [6,7]. The second case described a patient on continuous ambulatory peritoneal dialysis (CAPD) with peritoneal mucormycosis, which was also a rare manifestation described by only a small number of case reports worldwide [8]. The third and fourth cases were classic presentations of rhino- and rhino-orbital mucormycosis; one was COVID-19-associated mucormycosis (CAM) due to corticosteroid exposure in addition to suboptimally controlled diabetes mellitus, which was rarely reported among COVID-19 populations in Malaysia [4], followed by the last case with a typical risk factor of poorly controlled diabetes mellitus.

Challenges such as difficulty to culture the organism leading to a delay in diagnosis, limitation of antifungal choice due to the cost, adverse effects from antifungal therapy, as well as their outcome are being described in the following cases.

## Case presentation

Case 1

**Figure 1:**
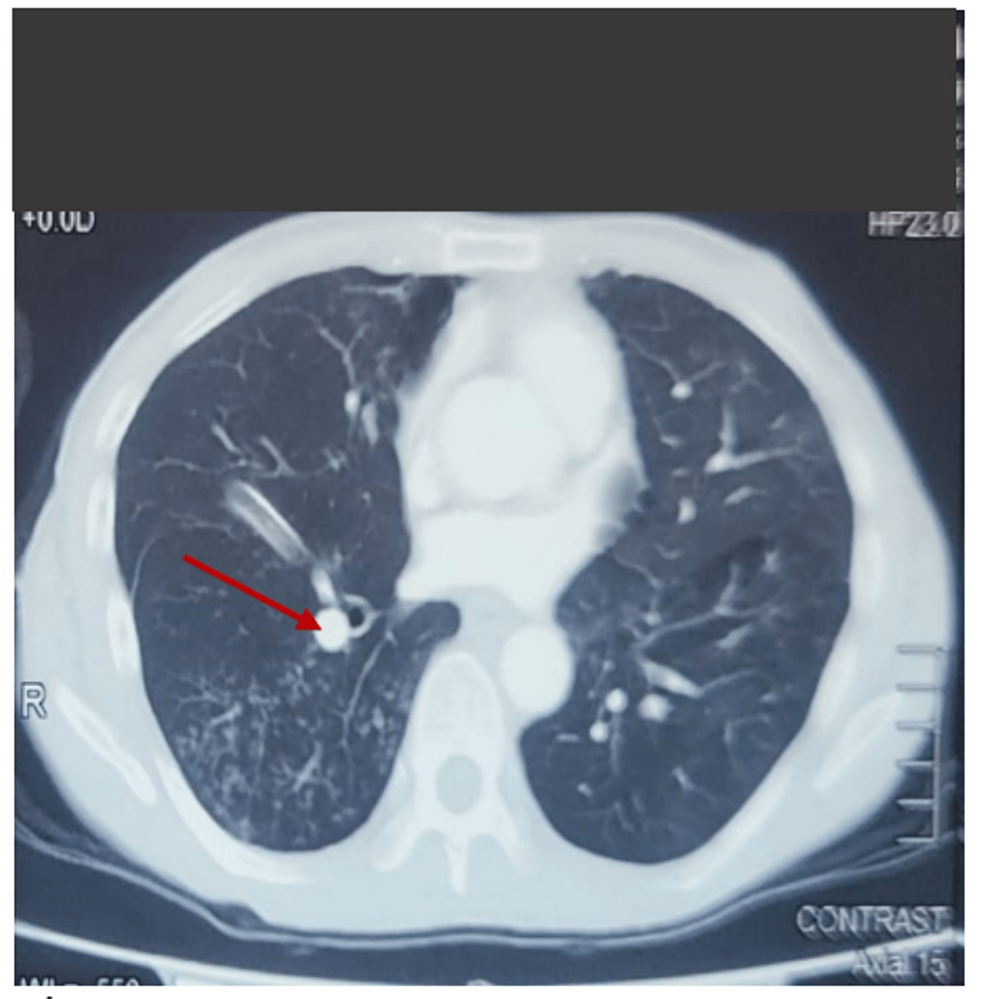
Contrast-enhanced computed tomography axial section showed a hyperdense lesion measuring approximately 1.3×1.4×1.9 cm (AP×W×CC) within the right bronchus with chronic fibrosis and cystic lung changes.

**Figure 2:**
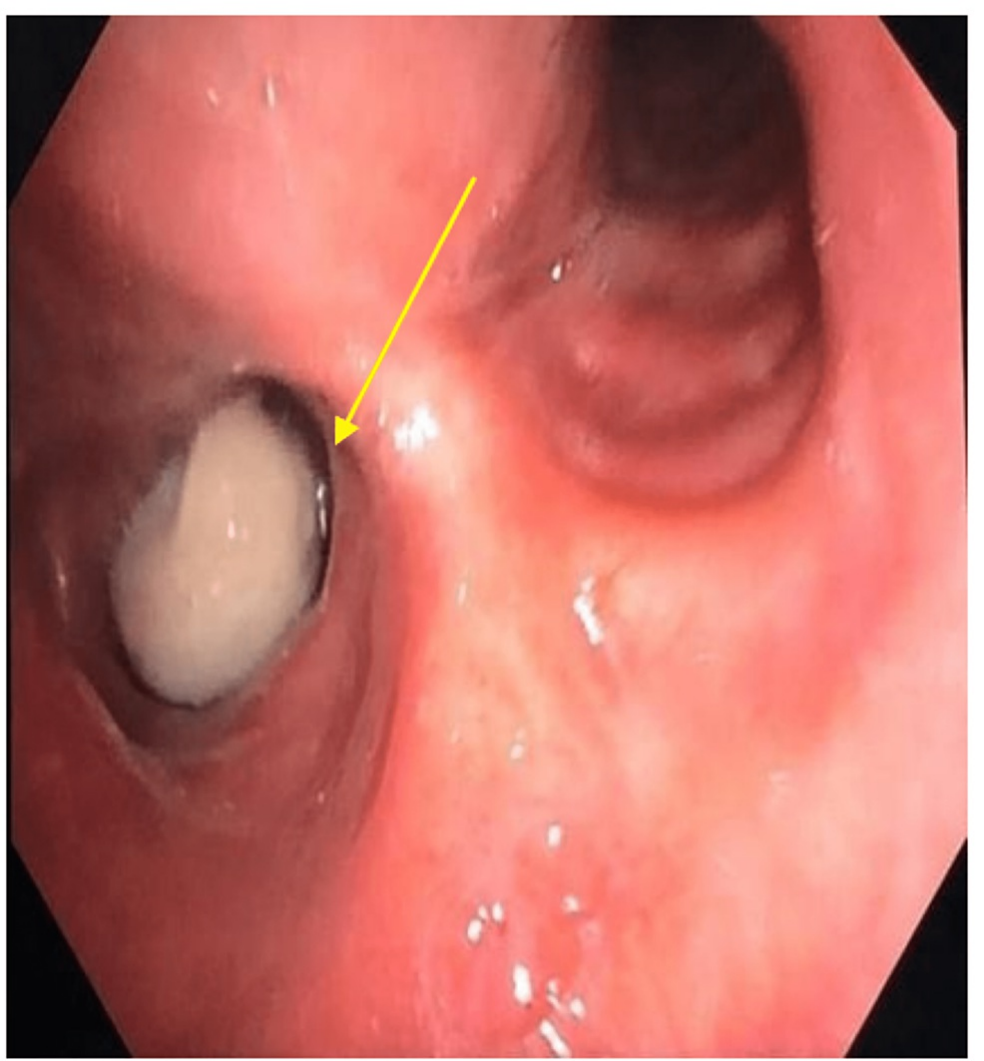
Bronchoscopy revealed a whitish round pedunculated mass over right bronchus intermedius.

A 56-year-old man with AIDS newly started on antiretroviral therapy (ART), untreated chronic hepatitis C coinfection, treated pulmonary tuberculosis and chronic obstructive pulmonary disease (COPD) classified as Gold E with exacerbations every two-monthly requiring a five-day course of prednisolone 30 mg once daily each episode had presented with fever, productive cough, and shortness of breath for four days. He was intubated due to bronchospasm, and a chest X-ray showed right apical pneumothorax with bilateral reticular shadowing seen over bilateral lung bases. White blood cell (WBC) count was 12.6×10^9^/L (normal range: 4-10×10^9^/L) and C-reactive protein (CRP) was 23.2 mg/L (normal range: <10.0 mg/L). Sputum direct smear was negative for acid-fast bacilli but endotracheal culture grew *Pseudomonas aeruginosa*. He was successfully extubated after five days of appropriate antibiotic and chest tube insertion but remained breathless on exertion. Contrast computed tomography (CT) thorax was done to rule out bronchopleural fistula due to persistent pneumothorax, which incidentally picked up a right bronchus intermedius endobronchial soft tissue lesion in a background of chronic lung changes (Figure 1). Bronchoscopy was done, showing a whitish round pedunculated mass over the right bronchus intermedius (Figure 2). Biopsy taken from the mass was negative for fungal culture, and histopathology reported necrotic, inflammatory debris as well as the presence of fungal bodies, which were mainly non-septate hyphae and yeasts, suggestive of mucormycosis with no features of malignancy or tuberculosis. There was a delay in treatment commencement due to initial negative culture with a provisional diagnosis of malignancy until the histopathology report was available. He was then initiated on intravenous (IV) isavuconazole 372 mg thrice daily for six doses, followed by 372 mg daily for a total of 10 days. Surgical intervention was deemed unsuitable due to poor lung reserve after a discussion with a thoracic surgeon. With treatment, his oxygenation normalised to 98% under room air, and eventually had his chest tube removed. The patient was subsequently discharged with oral posaconazole 300 mg daily. He was scheduled for an outpatient CT thorax for reassessment; however, he was readmitted for acute exacerbation of COPD during his second month of oral antifungal therapy and succumbed to death due to respiratory failure.

Case 2

**Figure 3:**
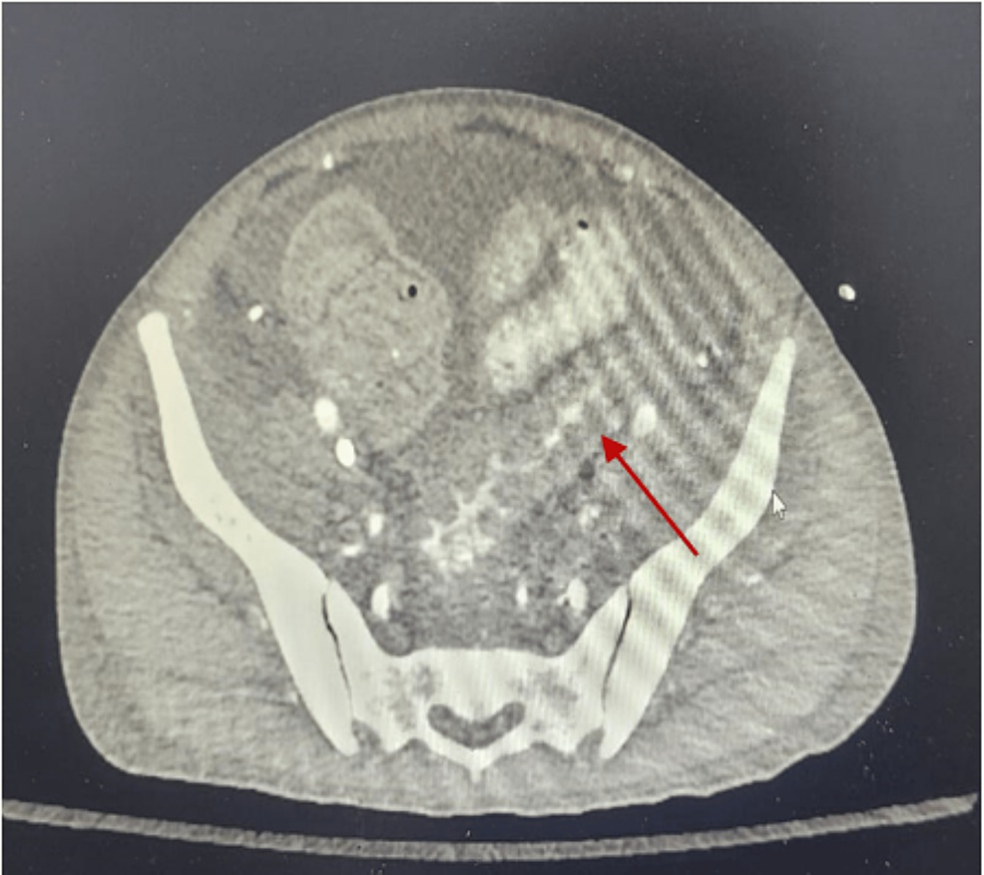
Contrast-enhanced computed tomography axial section showed thickening of peritoneal lining suggestive of peritonitis, colitis of distal large bowel and rectum with no signs of bowel perforation.

A 30-year-old man with HIV infection on ART for the past eight years with suppressed viral load, end-stage renal disease (ESRD) on CAPD, hypertension, and congestive cardiac failure presented with abdominal pain, cloudy peritoneal dialysate, and fever for a week. He was septic looking with generalised abdominal tenderness and guarding. WBC was 15.9×10^9^/L and CRP was 359.2 mg/L. He was initiated on intraperitoneal (IP) antibiotics for PD peritonitis but he continued to deteriorate and developed septic shock. Peritoneal fluid analysis showed the presence of 73 cells/mm^3^ with 100% polymorphonuclear leukocytes and culture grew *Cunninghamella bertholletiae*. CT abdomen showed peritonitis with colitis involving a long segment of the distal large bowel and rectum (Figure 3). Blood culture was negative. He was commenced on IV Amphotericin B (AmpB) deoxycholate 60 mg daily (1 mg/kg/day) and the Tenckhoff catheter was removed on day 10 of admission. Intraoperatively, 1.2 L of pus was drained from the peritoneal cavity, and slough was seen over the bowels, liver, stomach, and pelvic cavity. His condition improved and was out of high-dependency care initially but succumbed to death on day 30 of admission due to fluid overload.

Case 3

**Figure 4:**
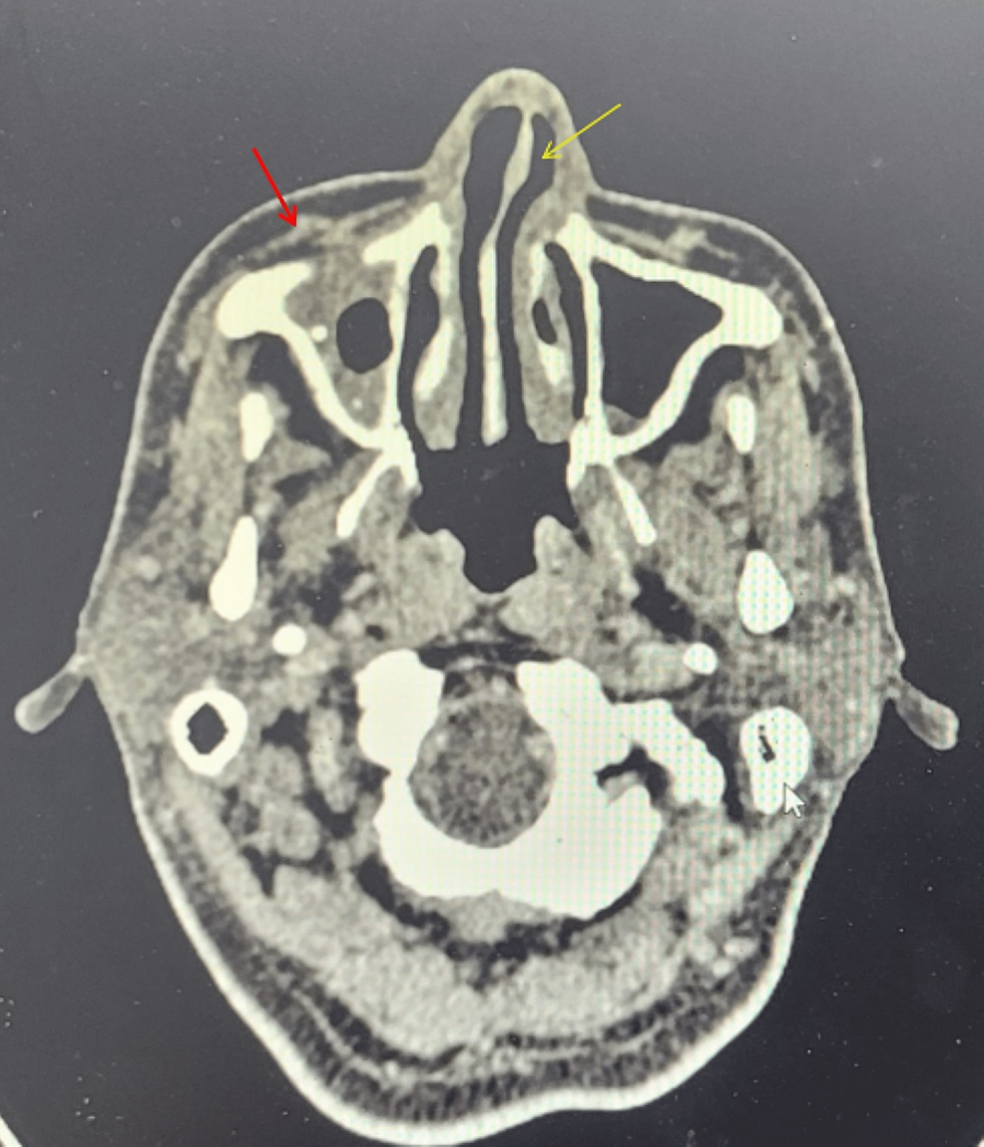
Computed tomography axial section showed median nasal septal deviation with opacification within the right maxillary sinus and destruction of the anterolateral wall of the right maxillary sinus.

A 62-year-old man with poorly controlled type 2 diabetes mellitus (T2DM), hypertension, single functioning kidney, ischemic heart disease, and a history of category 4 (National Institute of Health [NIH] classification) COVID-19 pneumonia treated with corticosteroids for six days in August 2021 presented with a non-healing wound over right maxillary arch and jaw pain after dental extraction in September 2021. WBC was 7.8×10^9^/L upon presentation. A contrast-enhanced CT brain and maxilla was done, showing median nasal septal deviation, right maxilla osteomyelitis, and involvement of the inferior wall of the right maxillary sinus (Figure 4). Incisional biopsy was done but both tissue fungal polymerase chain reaction (PCR) and culture were negative. Treatment was delayed until histopathology reported the presence of numerous broad ribbon-like, pauciseptate fungal hyphae, which confirmed the diagnosis of CAM. He was started on IV AmpB deoxycholate 80 mg daily (1 mg/kg/day) and had a surgical debridement done. Intraoperatively, complex bone extirpation of the right maxillary sinus was seen, with healthy surrounding soft tissue. Unfortunately, he developed acute kidney injury after one week of AmpB and was switched to oral posaconazole 300 mg daily. He continued to improve with treatment, and reassessment nasoendoscopy at the third month of antifungal therapy showed healthy mucosa with clear sinus, and no secretion or collection was seen. A repeat CT brain and maxilla did not show signs of new or progressive destruction of the maxillary sinus. He has completed posaconazole for six months and remains stable without new or recurrent symptoms.

Case 4

**Figure 5:**
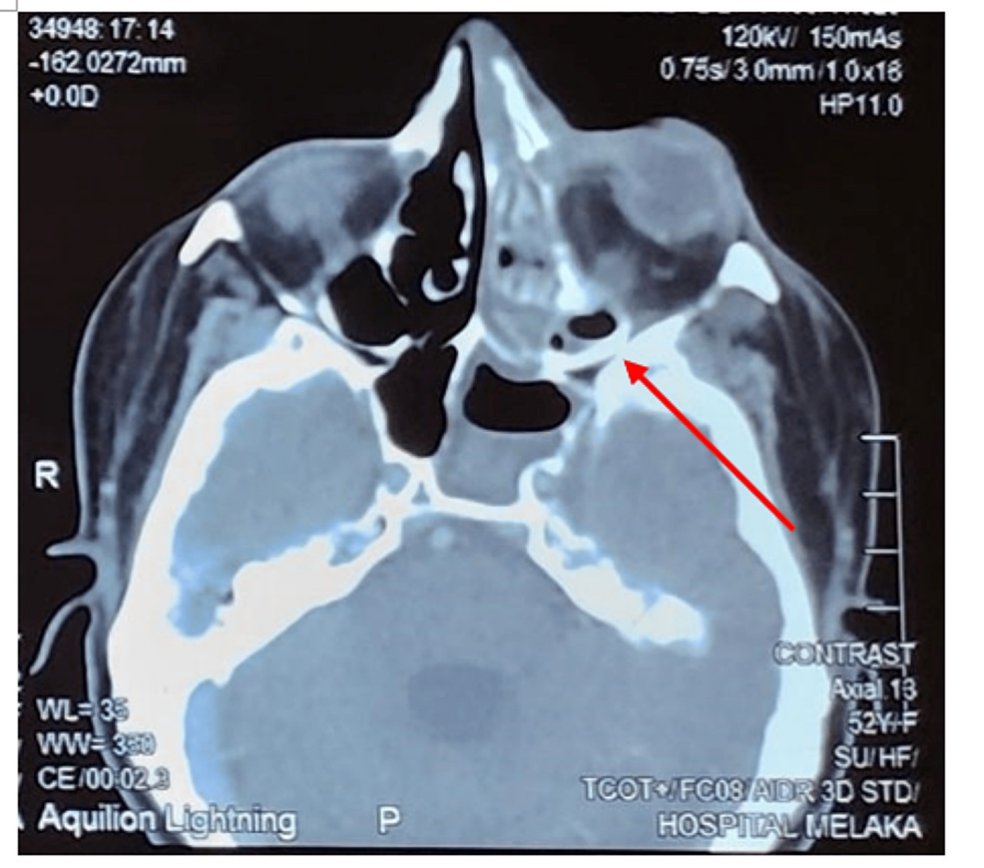
Contrast-enhanced computed tomography axial section showed mucosal thickening within the left frontal, ethmoid, maxillary, and sphenoid sinuses with involvement of the left orbit.

A 52-year-old lady with poorly controlled T2DM and a history of left above-knee amputation due to an infected left below-knee amputation stump presented with a one-week history of toothache, facial swelling, difficulty in opening her left eye, and fever. She was treated for periorbital cellulitis but did not improve with antibiotics. Clinical examination revealed a left hard palate necrotic patch with crusting seen over the left inferior turbinate, as well as the presence of left orbital apex syndrome and hazy cornea. CT brain and venogram showed left cavernous sinus and left superior ophthalmic venous thrombosis with mucosal thickening of all the ipsilateral sinuses (Figure 5). A provisional diagnosis of rhino-orbital invasive fungal disease was made; thus, she underwent endoscopic sinus surgery and debridement. Intraoperative incisional biopsy of the left palate grew *Rhizopus oryzae*. Histopathological examination showed a large area of abscess formation associated with extensive tissue necrosis, fungal bodies exhibiting pauciseptate, broad ribbon-like fungal hyphae suggestive of mucormycosis. Bacterial and fungal blood cultures were negative. She was treated with IV AmpB deoxycholate 80 mg daily (1 mg/kg/day). Unfortunately, she developed acute right-sided hemiparesis after two weeks of antifungal therapy due to new-onset left internal carotid artery arteritis picked up from a magnetic resonance imaging (MRI), secondary to the contiguous spread of the infection. A lumbar puncture was done to rule out cerebral involvement, and both cerebrospinal fluid (CSF) fungal PCR and culture were negative. The patient eventually had a left partial maxillectomy and insertion of a surgical plate with bilateral arcus zygomatic suspension wire followed by nasal and oral toileting, refashioning, and suturing of the left eye. Repeated intraoperative biopsy still showed occasional fungal bodies. Otherwise, she has completed six weeks of IV AmpB deoxycholate, remains asymptomatic, and is currently on the third month of oral posaconazole 300 mg daily (Table 1).

**Table 1:** Summary of the clinical manifestations, associated risk factors, culture and tissue diagnoses, treatment courses, and outcomes of all four cases. HIV, human immunodeficiency syndrome; COPD, chronic obstructive pulmonary disease; PTB, pulmonary tuberculosis; CCF, congestive cardiac failure; ESRD, end-stage renal disease; CAPD, continuous ambulatory peritoneal dialysis; HPT, hypertension; DM, diabetes mellitus; IHD, ischemic heart disease.

Case	Phenotype diagnosis	Comorbidities	Additional risk factor	Culture/fungal PCR	Histopathology	Treatment history	Treatment duration	Outcome
1	Pulmonary	HIV, chronic hepatitis C, COPD, history of PTB	Steroid use	No growth	Mucormycosis	Isavuconazole, posaconazole	10 weeks	Dead
2	Peritoneal	HIV, CCF, ESRD on CAPD, HPT	-	Cunninghamella bertholletiae	NA	AmpB deoxycholate	4 weeks	Dead
3	Rhino	DM (HbA1C 9.2%), HPT, IHD, single functioning kidney	Steroid use	No growth/PCR negative	Mucormycosis	AmpB deoxycholate, posaconazole	6 months	Alive
4	Rhino-orbital	DM (HbA1C 12.5%)	-	Rhizopus oryzae	Mucormycosis	AmpB deoxycholate, posaconazole	Ongoing (12^th^ week of treatment)	Alive

## Discussion

Mucormycosis is one of the common invasive mycoses in patients with haematological and allogeneic stem cell transplantation, as well as diabetic patients. As opposed to classical risk factors, HIV infection does not inherently predispose to mucormycosis unless associated with illicit IV drug use, neutropenia, diabetes mellitus, or corticosteroids, with an overall mortality rate of 52.2% [6]. This was also supported by Antorini et al*.,* which included 1630 autopsies of patients who died of AIDS, and only two patients had mucormycosis [7]. Our first case correlated well with the findings above as he had AIDS with a history of repetitive corticosteroid use due to COPD.

Pulmonary mucormycosis can present as bronchitis, bronchopneumonia, cavitary lesions, or more rarely endobronchial lesions, which can cause airway occlusion [9]. Interestingly, the right lung is more commonly involved than the left, and there is a predilection for the involvement of the upper lobes [10]. Diagnosis is often difficult due to non-specific presentation. Our first case highlighted the importance to raise clinical suspicion of fungal infection in immunocompromised patients with fever and respiratory symptoms refractory to antibiotic therapy, with a low threshold to perform an invasive diagnostic procedure. Traditionally, debulking surgery with systemic antifungal therapy is recommended, even for patients with isolated endobronchial disease. Although a cure has been reported with medical management alone, Lee et al. [11] documented a decrease in survival (55% vs. 27%) when antifungal therapy is not combined with surgical resection. Unfortunately, many patients might not be surgical candidates because of poor lung function as in our first case, or lesions that are not amenable to resection anatomically. Emerging treatment modality such as flexible bronchoscopic cryotherapy to facilitate ongoing antifungal treatment by decreasing the fungal burden has been described [12]. Daily endobronchial instillation of AmpB for the treatment of localized pulmonary mucormycosis has also been reported and showed promising outcomes. It may also allow shorter courses of systemic therapy with lower doses of the drug if adequate local concentrations can be demonstrated [13]. Further study to define the appropriate dose and duration of therapy, as well as complications such as inflammatory response causing endobronchial obstruction and toxicities are warranted as these methods are potentially a rope of hope for vulnerable patients to improve their odds of a nonsurgical cure.

Rhino-orbito-cerebral mucormycosis (ROCM) is the most common manifestation among poorly controlled diabetics and *Rhizopus oryzae* is responsible for nearly 90% of the cases, with an overall mortality rate of over 50% [14]. The incidence of mucormycosis has surged during the COVID-19 pandemic particularly in South Asian countries due to the usage of corticosteroids. Sinuses were the most common sites of mucormycosis among COVID-19 patients at 79.4%, with maxillary sinus (47.4%) being the most infected. The mean duration between the diagnosis of COVID-19 and mucormycosis was 16.15 days (range 2-90 days) [15]. However, it remains rare in Malaysia and only one case was reported [4]. Our third patient also had a history of category 4 COVID-19 pneumonia with a short course of corticosteroid use, as well as suboptimal controlled T2DM with HbA1C of 9.2% which were significant enough to predispose him to mucormycosis. Depending on the site and the extent of the disease, the prognosis varies. Vaughan et al. reported an overall survival of 59.5% with treatment and as low as 21% if left untreated [16]. Fortunately, our patient did not have intracranial or orbital involvement which could worsen his prognosis [17]. He responded well to surgical debridement and appropriate antifungal therapy.

Peritoneal mucormycosis is extremely rare, only described by 16 case reports worldwide [8]. The majority were related to CAPD, and the mortality rate in patients receiving delayed or inappropriate treatment has been as high as 60% [18], with common complications such as sclerosing peritonitis, adhesions with resulting bowel obstructions or stricture, invasion of the bowel wall, and abscess formation [19]. Intraperitoneal AmpB instillation is not routinely recommended due to local irritation and it can contribute to adhesion formation with subsequent loss of the peritoneum as a dialyzing membrane. A high index of suspicion in non-resolving peritonitis is required for diagnosing invasive fungal infections such as peritoneal mucormycosis, especially if cultures remain negative and the patient did not respond to antibiotic therapy. Early diagnosis of the fungi with the help of peritoneal dialysis effluent culture or peritoneal biopsy, rapid institution of antifungal therapy, and a low threshold for surgery are key parameters for a successful outcome of this group of patients [8].

Microscopy (direct or histopathology) and culture of clinical specimens are the cornerstones of diagnosing mucormycosis. However, the report suggested that only one-third of all microscopically positive specimens reveal positive cultures [20]. This low culture positivity may be attributed to different factors ranging from sample collection, storage to sample processing, which might affect the viability of Mucorales [21]. A study that recruited pulmonary mucormycosis cases among cancer patients reported that although not statistically significant, culture-positive patients were more likely to exhibit a high burden of necrosis and hyphae, but less vascular invasion compared to culture-negative patients. In terms of clinical characteristics, culture-positive patients were more likely to have haematological malignancy, a history of haematopoietic cell transplant, severe lymphopenia (absolute lymphocyte count ≤ 500/µL), and monocytopenia (absolute monocyte count ≤100/µL) [22]. Molecular-based diagnosis such as conventional polymerase chain reaction (PCR) may be a useful adjunct to facilitate an earlier diagnosis in culture-negative cases [21].

Treatment of mucormycosis is a combination of surgery, when possible, correction of underlying risk factors, and aggressive antifungal therapy [23]. International guidelines recommend using liposomal AmpB 5-10 mg/kg per day [23]. Renal function tests must be performed on patients before commencing amphotericin therapy. AmpB deoxycholate should be restricted to settings in which there is no other antifungal therapy due to more severe systemic side effects such as infusion-related reactions and nephrotoxicity [14]. It was used as the first-line treatment for most of our reported cases as other antifungals were not readily available in our centre. A retrospective cohort study reported that AmpB deoxycholate is still an acceptable alternative for treating mucormycosis in resource-constrained settings [24]. AmpB should be continued until the patient has shown signs of improvement, which usually takes several weeks. Till date, there is no conclusive data supporting combination therapy; hence, it is not recommended in the major treatment guidelines. For patients who have responded to liposomal AmpB, posaconazole or isavuconazole can be used as oral step-down therapy. Isavuconazole has shown similar efficacy to liposomal AmpB against mucormycosis [25]. The treatment course should be continued until there is a clinical improvement as well as resolution of radiographic signs of active disease. When feasible, it should also be continued until reversal of underlying immunosuppression has been achieved. Therapy often extends for months, and it is not surprising for certain patients to remain on lifelong therapy if immunosuppression cannot be corrected.

## Conclusions

In conclusion, our four cases depict the common presentations of mucormycosis such as pulmonary and rhino-orbital, as well as a rare manifestation such as peritonitis. Apart from traditional risk factors, we presented a case from the AIDS population, as well as a COVID-19-associated mucormycosis, which were both rare risk factors for mucormycosis. Diagnosis remains a challenge as not all microscopically positive specimens have positive cultures. Hence, clinicians should request urgent histopathology reporting if clinical suspicion of invasive fungal infection is high. Discussion with microbiologists and lab technicians to improve sample storage and processing in order to increase the yield of the organism is also crucial. Liposomal AmpB was not used as our first-line treatment, and a majority of our patients were treated with AmpB deoxycholate due to financial aspects. Despite that, our patients responded to the treatment, and the use of AmpB deoxycholate is also supported by evidence from resource-limited countries. Treatment duration varies from case to case, reflecting the effect of the different presentations on the patient's response as well as the adequacy of source control. In addition, further studies regarding local instillation of AmpB or cryotherapy for pulmonary mucormycosis are warranted as surgery is of high risk in this group of patients and they will benefit significantly from a combination of systemic antifungal therapy and local treatment. Finally, this case series aims to contribute to local epidemiology to estimate its prevalence, which could help draw stakeholder attention to early diagnosis of mucormycosis.
